# Goserelin-Induced Chemical Burn: A Case Report and Review of the Literature

**DOI:** 10.7759/cureus.45692

**Published:** 2023-09-21

**Authors:** Aditya Mahadevan, Brian Warnecke, Elaine Chiao, Nellie Nafissi, Kritisha Parajuli, Nejina Rijal, Ritesh Parajuli

**Affiliations:** 1 Department of Hematology and Oncology, University of California Irvine School of Medicine, Orange, USA; 2 Department of Hematology and Oncology, University of California Irvine Medical Center, Orange, USA; 3 Department of Medicine, Nepal Medical College and Teaching Hospital, Kathmandu, NPL; 4 Department of Medicine, Kathmandu Medical College, Kathmandu, NPL

**Keywords:** rash, ovarian suppression, aromatase inhibitor, chemical burn, goserelin

## Abstract

A chemical burn resulting from luteinizing hormone-releasing hormone agonists (LHRHa) is a rare adverse effect that has not been well-documented in prior literature. In this case report, we report a partial-thickness burn that developed following a single subcutaneous injection of goserelin. To our knowledge, this is the first description of goserelin-induced chemical burn in the literature. The importance of early identification and treatment of LHRHa-associated cutaneous reactions must be highlighted to ensure optimal oncologic management and patient comfort.

## Introduction

Aromatase inhibitors (AIs) have become a mainstay of breast cancer treatment, with clinical trials showing significant reductions in recurrence rate with aromatase inhibitors as compared to tamoxifen [1]. Luteinizing hormone-releasing hormone agonists (LHRHa), including goserelin, triptorelin, and leuprorelin, have emerged as well, achieving ovarian function suppression (OFS) through sustained suppression of the release of follicle-stimulating hormone (FSH) and luteinizing hormone (LH) from the pituitary [2]. While LHRHa initially produces a surge in ovarian hormones, long-term administration of LHRHa reduces ovarian hormone production and secretion by causing a downregulation and desensitization of LHRH receptors in pituitary gonadotropic cells, resulting in a reduction of circulating estrogens that slows the growth of hormone receptor-positive tumors [2,3]. While there is conflicting evidence regarding the benefits of adding OFS (LHRHa) to endocrine therapy, the most recent available evidence suggests that OFS added to either tamoxifen or AIs can provide significant benefit in premenopausal patients with less favorable clinicopathological characteristics, such as those who have received previous chemotherapy [2]. 

Despite its benefits, endocrine therapy does have several toxicities. Tamoxifen has several documented side effects, most commonly vulvovaginal and vasomotor symptoms like hot flashes, while also increasing the risk of thromboembolic events and endometrial cancer [4]. Aromatase inhibitors, in contrast, tend to cause arthralgias and bone loss [4]. While ovarian suppression in premenopausal women has not been shown to significantly impact fertility, ovarian suppression leads to a significantly higher incidence of hot flashes and vaginal dryness [5,6].

Cutaneous reactions are a relatively uncommon side effect of LHRHa, occurring in approximately 6.3% to 11.1% of patients who underwent clinical trials that included an LHRHa [7-9]. There are recent case reports of anaphylaxis and erythema nodosum in response to goserelin infusion [10,11]. Subcutaneous granuloma formation at the injection site of leuprorelin acetate has also been documented [12]. We present a unique case of a chemical burn reaction following a single infusion of goserelin. 

## Case presentation

This is a 37-year-old pre-menopausal patient with a past medical history of Tourette syndrome, attention-deficit/hyperactivity disorder, and migraines. In November 2022, she was noted to have a left breast mass. A mammogram revealed a 1.5 cm left breast mass at the one o'clock position, and no lymph nodes were radiographically identified. A core needle biopsy revealed invasive ductal carcinoma grade 2, estrogen and progesterone positive, human epidermal growth factor receptor 2 (HER2) negative. Positron emission tomography (PET) CT showed no evidence of metastasis. She did not require neoadjuvant chemotherapy. A lumpectomy and sentinel lymph node dissection were performed. Pathology revealed a left-sided 1.9 cm, grade 3 invasive ductal carcinoma. The tumor was stained 91-100% estrogen receptor (ER), progesterone receptor (PR) positive. HER2 was negative by immunohistochemistry. Ki-67 was 25%. The tissue specimen demonstrated focal lymphovascular invasion and extracapsular extension. One of three biopsied lymph nodes showed evidence of invasive ductal carcinoma. Altogether, her cancer was staged as IIA (pT1cN1aM0). 

Based on her tissue specimen, an Oncotype Dx score of 21 was obtained, consistent with intermediate risk. The patient agreed to proceed with adjuvant chemotherapy with six cycles of cyclophosphamide and docetaxel. She was referred for oocyte preservation, and her eggs were harvested prior to chemotherapy administration. Endocrine therapy and ovarian suppression were planned, given her pre-menopausal status and hormone receptor positivity. She was initially treated with a single subcutaneous injection of 3.6 mg goserelin. Of note, she also received a pegfilgrastim injection at the same infusion center visit. 

**Figure 1:**
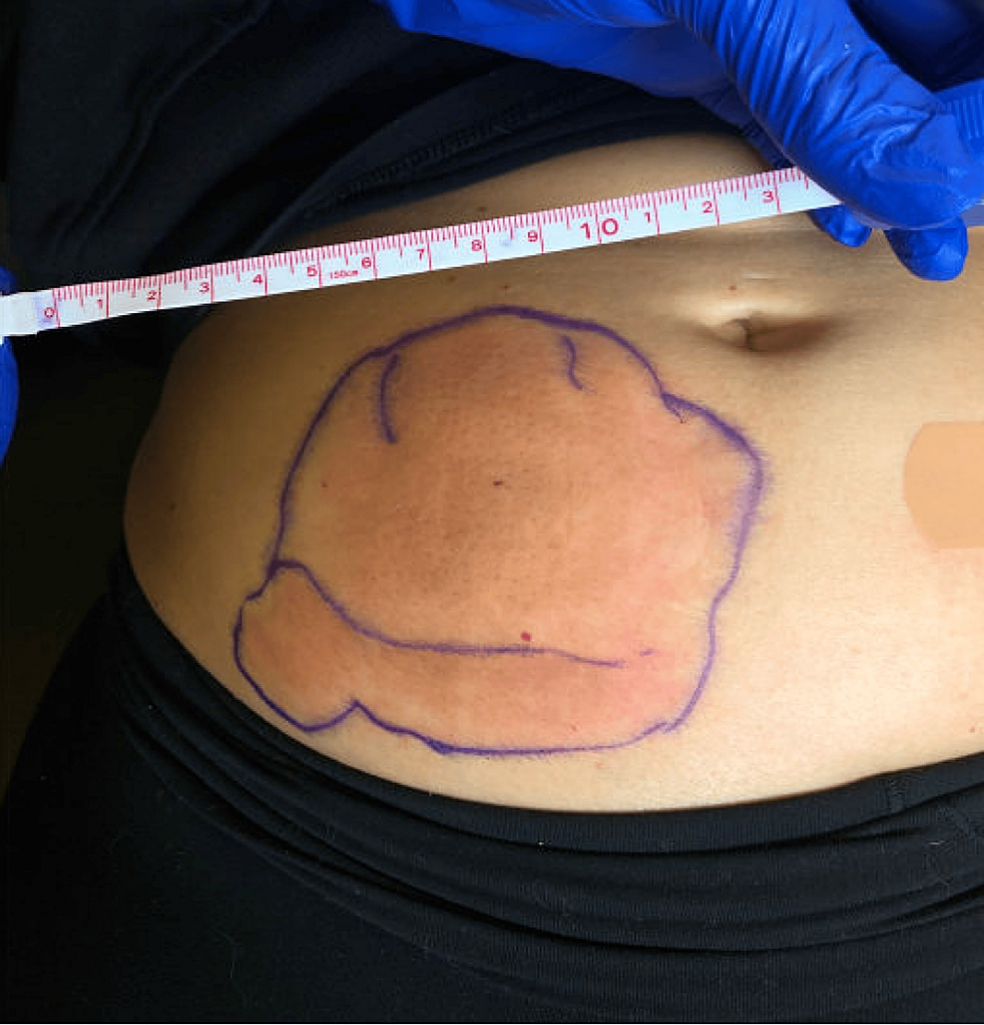
9x10 cm region of erythema and induration on the right lower abdominal wall

One hour after her initial dose of goserelin, a 9 cm by 10cm region of erythema and induration developed surrounding the subcutaneous injection site on her right lower abdominal wall (Figure 1). At the time of onset, the lesion was non-tender and non-pruritic. She received 25 mg diphenhydramine and 100 mg hydrocortisone with symptomatic relief and was discharged in stable condition.

**Figure 2:**
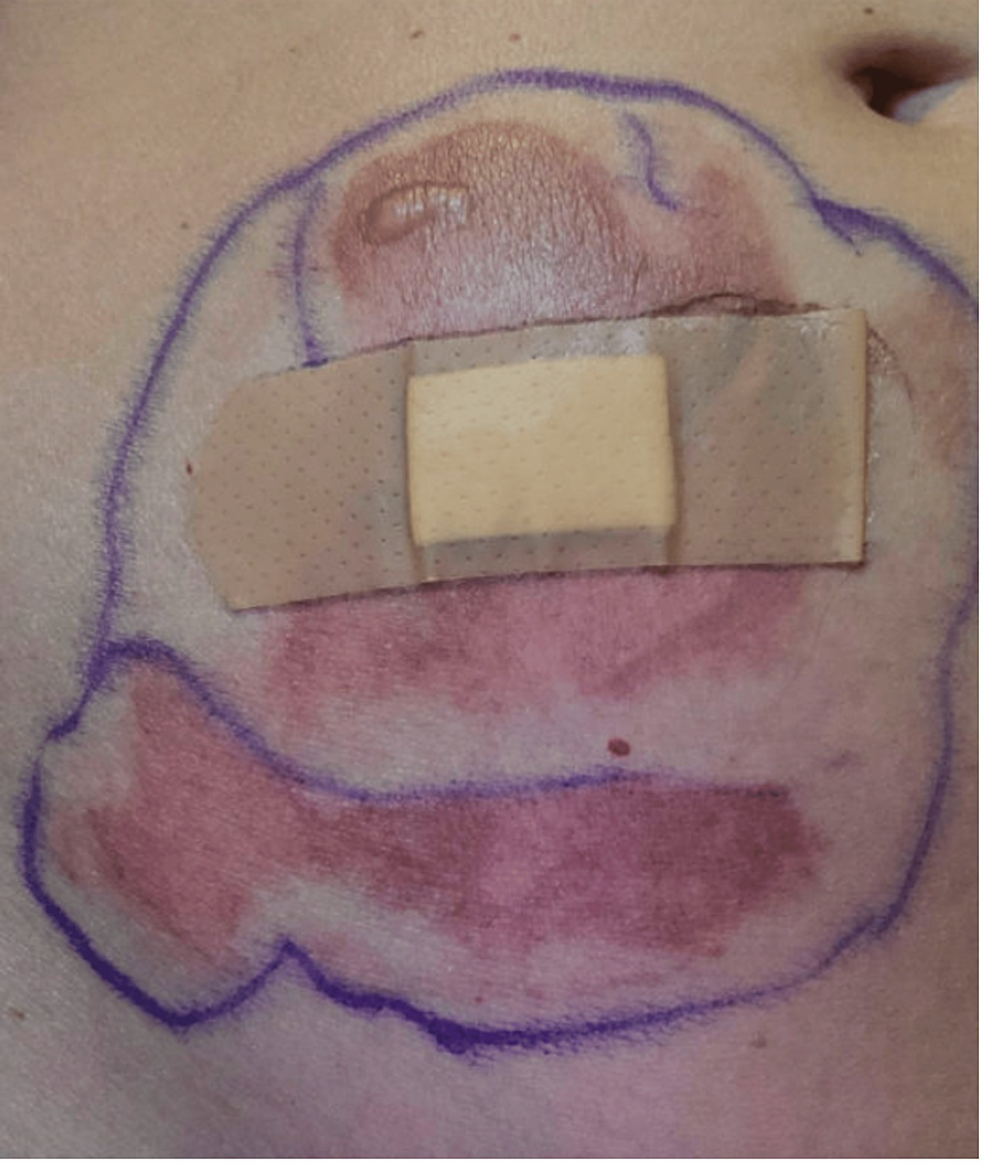
9x10 cm area of erythema with small overlying fluid-filled vesicle

The following day, the rash had progressed to painful blisters, and her skin began to peel (Figure 2). Dermatology was consulted, and felt the rash to be a partial-thickness burn based on the presence of erythema and blistering. Symptomatic treatment was recommended. Subsequently, goserelin was discontinued due to this adverse side effect, and the patient was transitioned to leuprolide. Over the next several weeks, the burn improved without the need for a skin graft or steroids. 

## Discussion

Upon initial examination of the skin rash, potential differential diagnoses included an allergic drug reaction, contact dermatitis, injection-related bacterial cellulitis, or other infectious etiology. In comparison to the site of goserelin injection, the pegfilgrastim injection site appeared normal despite being given at the same time as goserelin, indicating the reaction was likely due to goserelin. Skin rash is a common manifestation of drug-induced allergies, and severe cases can involve angioedema of the face, lips, and tongue or life-threatening anaphylaxis. Given that these symptoms were not appreciated in this patient, an allergic drug reaction was thought to be unlikely. Furthermore, the absence of urticaria and fluid-filled blisters made contact dermatitis and other hypersensitivity reactions less likely. The patient's acute onset of symptoms, well-demarcated area of erythema and blistering, and lack of constitutional symptoms were less consistent with cellulitis. 

Based on the overall clinical presentation, including lack of pruritis, rash, and systemic symptoms, and the presence of erythema and painful blisters, the injection-site reaction was deemed to be a second-degree chemical burn. The pathophysiology of chemical burns constitutes a series of reactions that occur when the skin or underlying tissue comes into contact with an irritating or corrosive substance, leading to one of six mechanisms: oxidation, reduction, corrosion, protoplasmic poisons, vesicants, and desiccants [13]. Shared among these reactions is the presence of protein denaturation, tissue damage, vascular change, and subsequent inflammatory response [13].

Chemical burns broadly fall under the categorization of injection site reactions, a cutaneous phenomenon of various chemotherapeutic agents, collectively known as toxic erythema of chemotherapy (TEC) [14]. While the mechanism is not fully understood, the pathophysiology of TEC may involve direct toxic effects on the epidermis and eccrine epithelium as well as the release of granulsyin by natural killer cells [15-17]. Glatiramer acetate has been described as a culprit behind related skin syndromes such as allergic rash, flushing, skin swelling, and rare cases of erythema nodosum [18]. To our knowledge, there is no prior published literature reporting TEC or chemical burns secondary to LHRHa. Given the presence of an erythematous plaque accompanied by blistering, however, our patient's lesion was consistent with goserelin-induced TEC [14].

The patient's tenderness and erythema without associated symptoms of warmth or itching were consistent with a grade 1 injection site reaction [18]. The absence of edema, phlebitis, ulceration, and necrosis made grades 2-5 toxicity less likely. Grade 1 reactions are typically mild in nature and do not necessitate extensive treatment. In most cases, conservative measures such as warm compresses and over-the-counter analgesics can effectively alleviate pain intensity and duration associated with injection site reactions [19]. Consistent with this assertion, our patient's cutaneous symptoms improved substantially with conservative management. Repeat skin examination at a subsequent clinic visit revealed significant clinical improvement.

## Conclusions

This case report describes the development of a partial-thickness burn following a single subcutaneous infusion of goserelin, which later resolved following treatment. This case report adds to the small body of literature describing dermatological adverse events resulting from endocrine therapy. While a goserelin-induced chemical burn has not been documented previously, early recognition and treatment of this cutaneous reaction is essential to ensure optimal management and patient comfort. 
